# Murder Committed by a Patient upon His Medical Adviser

**Published:** 1853-01

**Authors:** 


*Murder Committed by a Patient upon his Medical Ad viser.*—The list of the fearful casualties to which medical practitioners are exposed is indeed extensive, and much more so than the public dream of. Here is a shocking instance of a surgeon falling a sacrifice to the mental aberration of his patient. The murderer—Paul Neunez —is twenty-six years old, son of the post-master of a village called La Janette, in Belgium, and had for the last two years been under the care of Dr. Leclerq, residing at Hal, a place situated at a short distance off La Janette. Neunez was affected with cerebral disease, and Dr. Leclerq had frequently warned the mother that her son was not right about the head, and that he should be watched. The opinion of the doctor's annoyed the patient very much; he often complained of him, and conceived at last such a hatred of his medical attendant, that he resolved to kill him. He went therefore to Brussels, August 15, where he bought a double-barrelled pistol, which he made the gunsmith load for him with two bullets; he provided himself with powder and some more balls and returned home. On the 6th of September he went to Hal, during the fair, intending to shoot Dr. Leclerq at a public ball, but the latter was prevented from going. The deed was therefore put off; but on the Sunday following, Neunz returned to Hal with the determination of using his pistol at the door of the church. He inquired of a man living opposite at what time the doctor generally went to mass, and having been told that the latter was not in the habit of going, he said, "So much the worse for him, I must go to his house then." He went straightway, and was admitted by the male servant and Mrs. Leclerq, with whom he conversed freely, whilst waiting to be introduced into the doctor's study. After consulting with his unfortunate medical adviser for a few moments (the latter having received him with great kindness,) Dr. Leclerq proceeded to write a prescription, when Neunez quietly walked up to him, placed the pistol on the victim's left ear, and fired. A slight struggle ensued between the wounded man and his assassin, but the latter covered with blood, soon released himself, and walked out of the house before Mrs. Leclerq or the domestics had time to rush to the study whence the report had proceeded. The murdered man had attempted to follow Neunez, had left his study, and was found lying speechless and covered with blood on the threshold of his dining-room, where he soon expired. The wretched culprit went, after this fearful deed, to an inn apposite Dr. Leclerq's house, and assured the inmates, who were terrified at his being covered with blood, that they need not be alarmed—it was only the blood of that doctor, whom he had just killed; and asked for water to wash himself. When examined by the magistrate, he said that it was himself who had shot Dr. Leclerq, and that he hoped he was dead, as, being now in prison, he could not go and finish him. When the unfortunate wife of his victim was mentioned to him, he said that he was very sorry for her, but that, as he had property, he would will it to her before he was guillotined.—*London Lancet.*

